# Record-linkage comparison of verbal autopsy and routine civil registration death certification in rural north-east South Africa: 2006–09

**DOI:** 10.1093/ije/dyu156

**Published:** 2014-08-21

**Authors:** Jané Joubert, Debbie Bradshaw, Chodziwadziwa Kabudula, Chalapati Rao, Kathleen Kahn, Paul Mee, Stephen Tollman, Alan D Lopez, Theo Vos

**Affiliations:** ^1^Burden of Disease Research Unit, South African Medical Research Council, Parow Vallei, Western Cape, South Africa, ^2^School of Population Health, The University of Queensland, Brisbane, QLD, Australia, ^3^MRC/Wits Rural Public Health and Health Transitions Research Unit (Agincourt), School of Public Health, University of the Witwatersrand, Johannesburg, South Africa, ^4^Umeå Centre for Global Health Research, Department of Public Health and Clinical Medicine, Umeå University, Umeå, Sweden, ^5^INDEPTH Network, Accra, Ghana, ^6^Melbourne School of Population and Global Health, The University of Melbourne, Carlton, VIC, Australia and ^7^Institute of Health Metrics and Evaluation, University of Washington, Seattle, USA

**Keywords:** Mortality, data quality, causes of death, vital statistics, verbal autopsy, data linkage, Agincourt Health and Demographic Surveillance System, Statistics South Africa, rural South Africa

## Abstract

**Background:** South African civil registration (CR) provides a key data source for local health decision making, and informs the levels and causes of mortality in data-lacking sub-Saharan African countries. We linked mortality data from CR and the Agincourt Health and Socio-demographic Surveillance System (Agincourt HDSS) to examine the quality of rural CR data.

**Methods:** Deterministic and probabilistic techniques were used to link death data from 2006 to 2009. Causes of death were aggregated into the WHO Mortality Tabulation List 1 and a locally relevant short list of 15 causes. The matching rate was compared with informant-reported death registration. Using the VA diagnoses as reference, misclassification patterns, sensitivity, positive predictive values and cause-specific mortality fractions (CSMFs) were calculated for the short list.

**Results:** A matching rate of 61% [95% confidence interval (CI): 59.2 to 62.3] was attained, lower than the informant-reported registration rate of 85% (CI: 83.4 to 85.8). For the 2264 matched cases, cause agreement was 15% (kappa 0.1083, CI: 0.0995 to 0.1171) for the WHO list, and 23% (kappa 0.1631, CI: 0.1511 to 0.1751) for the short list. CSMFs were significantly different for all but four (tuberculosis, cerebrovascular disease, other heart disease, and ill-defined natural) of the 15 causes evaluated.

**Conclusion:** Despite data limitations, it is feasible to link official CR and HDSS verbal autopsy data. Data linkage proved a promising method to provide empirical evidence about the quality and utility of rural CR mortality data. Agreement of individual causes of death was low but, at the population level, careful interpretation of the CR data can assist health prioritization and planning.

**Key Messages**

- Civil registration (CR) is a well-established official national system in South Africa, and mortality information from CR is a key data source that can guide health prioritization. However, studies continue to indicate quality problems with cause-of-death information.
- This first study, linking mortality data from CR and from the Agincourt Health and Demographic Surveillance System, demonstrates that data linkage between these sources is possible.
- The study offers the first empirical evidence of the extent and diversity of misattribution of HIV deaths in CR data from a rural setting in South Africa.
- Urban and national findings of systematic biases in CR cause-of-death data are confirmed by this rural study, pointing to the countrywide urgency to improve CR cause-of-death data.
- In the interim, however, it is encouraging that the confirmed biases can facilitate adjustment of cause profiles after careful interpretation to better inform rural health prioritization.

## Introduction

Reliable and valid mortality data are key inputs for appropriately aligning a population’s health care delivery with its health care needs. However, there is a lack of such information in many low- and middle-income countries, with particular limitations in sub-Saharan Africa.^1–3^ Data for South Africa, an upper-middle income country, were categorized by the World Health Organization (WHO) in the group of countries with unsatisfactory levels of completeness of death registration and low-quality cause-of-death information.^3^^,^^4^ Since these assessments, a number of national initiatives have focused on improving completeness levels and quality of cause-of-death statistics from civil registration (CR).^5^^,^^6^ South African CR has national, all-inclusive geographical and population coverage as mandated by the Births and Deaths Registration Act of 1992 and subsequent amendments. Completeness of adult death registration was estimated to be 90% in 2000,^7^ and 89% and 78%, respectively, for infants and children under 5 years of age by 2006.^8^ Moreover, cause-of-death information from routine death registration is produced satisfactorily by Statistics South Africa (Stats SA) in terms of timeliness and sub-national availability.^9^ Whereas South African CR data are a key source for national health priorities,^10^ and are used to estimate levels and causes of mortality in many sub-Saharan African countries^11^ where there is little mortality information elsewhere,^1^ death registration remains incomplete,^8^^,^^12^ and studies continue to indicate quality problems with cause-of-death information.^13–16^ Cause quality is particularly compromised by the under-recording of HIV/AIDS as an underlying cause as found by reviewing medical records^13^^,^^14^^,^^17^ and as indicated by examining the plausibility of national age distributions for HIV-recipient or -indicator conditions.^15^^,^^16^^,^^18^

In addition to the annual cause-of-death reports produced from CR by Stats SA,^19^ cause-of-death data are collected at three South African study sites of the International Network for the Demographic Evaluation of Populations and Their Health (INDEPTH).^20–22^ Deaths have been monitored since 1992 at the Agincourt Health and Socio-demographic Surveillance Site (Agincourt HDSS), the oldest of these sites, using a verbal autopsy (VA) tool to establish the probable cause of death.^20^ However, no study to date has attempted to link mortality data from CR and an HDSS to assess completeness of rural death registration in the CR system, or the quality of CR cause-of-death data in a rural area in South Africa.

Linking mortality data from lay reports in VA with mortality data from medical certification in routine civil registration may pose challenges due to data confidentiality and the particular methods used to ascertain information surrounding the cause of death. In addition, in the absence of ‘gold standard’ sources such as post-mortem pathological autopsies, it may not be straightforward to decide on the source of data that will best serve as a reference standard. Quality problems with CR cause-of-death information continue to be reported, as referenced above. Simultaneously, the limitations of deriving causes of death from a VA approach are acknowledged.^23^ However, a number of studies point to plausible, valid and reliable cause-of-death results from VAs in the Agincourt HDSS. These include a local validation study comparing Agincourt HDSS VA diagnoses from 1992 to 1995 against clinician-derived diagnoses from hospital records, which found that the VA diagnoses closely approximated to that of the hospital records, with high sensitivity, specificity and positive predictive values for injuries and infectious and parasitic diseases, and reasonable accuracy for non-communicable diseases.^24^ Other studies detected plausible time trends in HIV-related mortality from physician-certified VA diagnoses in Agincourt,^25–27^ which correspond closely to the patterns estimated in the National Burden of Disease Studies.^18^^,^^28^ Furthermore, for 6153 Agincourt deaths from 1992 to 2005, using two very different approaches to cause-of-death analysis and attribution (i.e. physician-assessment vs probabilistic modelling with the InterVA model) closely comparable results were found for the major causes of death in the area: over the 14-year period, both approaches present closely similar results from VAs with increasing domination by HIV-related mortality, combined with large numbers of injury deaths, and relatively low non-HIV-related infectious disease mortality. The 10 leading causes accounted for 83% and 88% of all deaths, according to physician and probabilistic interpretation, respectively, with 8 of the 10 leading causes common to both methodologies.^29^ Another study focused on physician vs InterVA assessment for HIV-related deaths in the period 1992–2005,^25^ and a remarkably similar development of HIV-mortality over time was reported, estimating the overall HIV-mortality rate at 18.4% and 18.6% under physician and modelled interpretations, respectively.^25^ Moreover, considerable agreement over time in the five leading causes by age was found for the period 1992–2005.^26^

Against this background, we aimed to link and compare mortality data from the national CR system and the Agincourt HDSS, for the same individuals who died during the period 2006–09. Our objectives were: to quantify the level of completeness of death registration in the CR system; to compare leading causes of death from each data source; and to quantify the level of agreement of cause attribution between CR and VA data; in order to provide empirical evidence about the quality and utility of rural CR cause-of-death data.

## Methods

### Study setting and data sources

**Figure 1.**
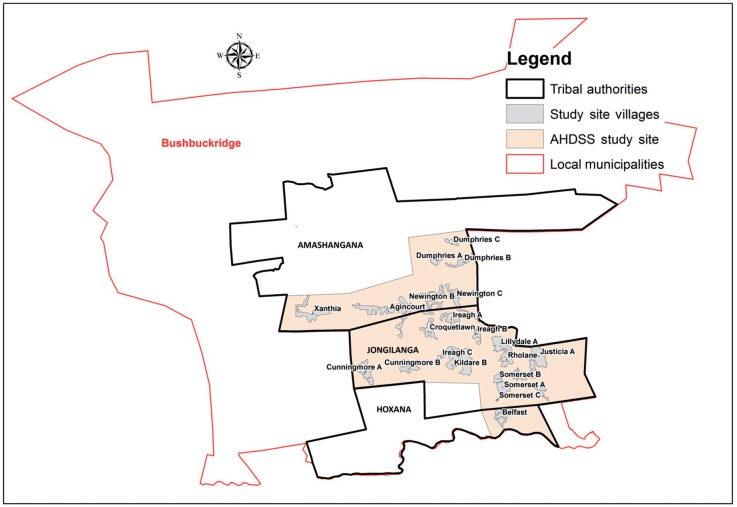
Agincourt HDSS villages within the study site borders, stretching across three tribal areas: Amashangana, Jongilanga and Hoxana, within the Bushbuckridge Municipality. Source: GIS section, Agincourt HDSS, created by Paul Mee.

The Agincourt HDSS, located in the Bushbuckridge district of Mpumalanga province in rural north-east South Africa (Figure 1), had a population of 87 000 people in 2009. The study site covers 420 km^2^, comprising 27 villages with limited development infrastructure, serviced by two health centres and six primary health care clinics within the site, and three district hospitals located 25 to 60 km away.^20^^,^^26^

A baseline population enumeration in 1992 has been followed by annual updates of resident status and vital events. For all deaths, trained fieldworkers interview the closest carer of the deceased to elicit signs and symptoms of the illness or injury preceding death, using a locally-validated, local-language VA instrument. Two medical doctors independently review the VA information and assign probable immediate, contributory and underlying causes using ICD-10 conventions. When a consensus cause cannot be reached, a third clinician, blind to earlier findings, assesses the details. The cause is coded ‘undetermined’ if an agreement cannot be reached.^20^^,^^24^

### National civil registration system and death registration

The Births and Deaths Registration Act requires a clinician to complete the death notification form (Form BI-1663) that includes the ICD recommended format for reporting the immediate, antecedent, underlying and contributory causes of death.^30^ For deaths in health facilities, attending or on-duty clinicians complete the form. For natural deaths at home, the deceased is taken to a morgue by undertakers who arrange for a clinician to examine the deceased and complete the form. In such instances, insufficiently available medical information about the deceased is commonly supplemented by information from relatives.^31^^,^^32^ When a clinician is not available, as may happen in some remote rural areas, a Death Report (From BI-1680) must be completed by an authorized traditional leader to certify the death and describe the circumstances around it.^31^^,^^32^ Approximately 10% of deaths are certified in this way.^33^ Unnatural deaths are subject to medico-legal investigation pursuant to the terms of the Inquests Act of 1959, and the deceased is taken to a government morgue where an autopsy is conducted. For death registration, the notification is submitted to a regional office of the Department of Home Affairs. All forms are subsequently compiled at national level, and then delivered to Stats SA where trained nosologists code causes of death to ICD-10 three-digit codes.^30^ They then determine the underlying cause of a death using the Automated Classification of Medical Entities software (ACME 2000.05).^34^

### Data extraction and linkage

Following ethical undertakings regarding the confidentiality and security of data, relevant mortality and birth data for the period 1 January 2006 to 31 December 2009 were extracted from the Agincourt HDSS database and linked with CR data at the premises of Stats SA. Eleven common variables were used for matching these death records: national identity number (a unique 13-digit number assigned to South African citizens), surname, sex, day of birth, month of birth, year of birth, day of death, month of death, year of death, village name and institution/venue where the death took place. Information for individuals who were either born, resident or died in the Bushbuckridge Municipality was extracted from Stats SA’s CR database. From this pool, records were extracted if the deceased was either born, resided or died in one of the tribal areas in which the Agincourt HDSS is located, i.e. Amashangana, Jongilanga and Hoxana (Figure 1).

Deterministic and probabilistic record linkage approaches were applied to find matches using routines implemented in T-SQL, the proprietary implementation of the SQL standard in the SQL*Server^TM^ software package.^35^ An anonymized, de-identified data set was created, including an indicator for matching status. The linkage methodology has been detailed elsewhere.^36^

### Data analysis

Stata 12^37^ and Microsoft Excel 2010 were used. The matching rate was calculated using the proportion of total Agincourt HDSS records that could be matched to CR records. The rate was compared with the proportion of HDSS records in which it was reported that the death had been registered in the CR system.

Causes from both sources were aggregated into the 103 causes of the WHO Mortality Tabulation List 1 (WHO list),^38^ used in similar studies elsewhere.^39^^,^^40^ Causes not coded according to standard ICD conventions, were recoded: one CR case assigned U51 (extensively drug-resistant tuberculosis) was recoded to A16 (respiratory tuberculosis); 10 VA cases assigned S and T codes (certain consequences of external causes) to Y34 (undetermined injury); and four VA cases assigned Z codes (factors influencing health status and contact with health services) to R99 (other ill-defined and unspecified natural causes).

To make the analyses more relevant to the local mortality burden, causes were further aggregated into a short list with 15 causes/cause groups (Table 1). Considering the use of pseudonyms for HIV deaths as reported in previous studies,^13–15^ investigating the age pattern of these deaths and examining the immediate, antecedent and contributory causes where available, 130 CR cases from ICD-10 codes B33 (other viral diseases), B45 (cryptococcosis), B59 (pneumocystosis), C46 (Kaposi sarcoma) and D84 (other immunodeficiencies) were recoded to B24 (HIV-disease). Additionally, based on previous findings,^15^^,^^16^ clinical advice, careful examination of age patterns and taking into account other causes attributed to these deaths, 21 CR cases from E86 (volume depletion) and E87 (other disorders of fluid, electrolyte and acid-base balance) were recoded to R99; 32 CR cases from K52 (other non-infective gastroenteritis and colitis) to A09 (other gastroenteritis and colitis of infectious and unspecified origin); and one CR and two VA cases from A39 (meningococcal infection) were recoded to G03 (meningitis).

**Table 1.** WHO Mortality Tabulation List 1 and ICD-10 codes for the short list of causes of death

Short list conditions	WHO Mortality Tabulation List 1 codes	ICD-10 codes
1 Diarrhoea	1-003	A09
2 Tuberculosis	1-005, 1-006	A15-A16
3 HIV disease	1-020	B20-B24
4 Remaining infectious & parasitic disease	1-002, 1-004, 1-007 to 1-010, 1-012 to 1-025	All remaining Ch A & B codes
5 Neoplasms	1-026	C00-D48
6 Diabetes	1-052	E10-E14
7 Meningitis	1-011, 1-059	A39, G00, G03
8 Hypertensive disease	1-066	I10-I14
9 Remaining heart disease	1-065, 1-067, 1-068	I00-I09, I20-I25, I26-I51
10 Cerebrovascular disease	1-069	I60-I69
11 Acute lower respiratory infections	1-073 to 1-075	J10-J11, J12-J18, J20-J22
12 Other respiratory disease	1-076 to 1-077	J40-J47, J30-J39, J60-J98
13 Symptoms & ill-defined conditions	1-094	R00-R99
14 External causes	1-095	V01-Y89
15 Remaining natural causes	All remaining codes	All remaining codes

We could not make use of ‘gold standard’ sources such as post-mortem pathological autopsy or expert review of hospital records against which to validate causes of death, as only 2% of CR deaths were autopsy ascertained, and less than half occurred in hospital. Instead, as done in a study in rural China,^41^ we used physician-certified VA causes of death as the reference diagnoses against which to examine the plausibility of the CR diagnoses. In spite of acknowledging the limitations of deriving causes of death from a VA approach,^23^^,^^42^ our decision was informed by the studies referred to in the Introduction,^18^^,^^24–29^ which lend support to the quality of Agincourt VA cause-of-death data and strengthen our confidence in VA causes of death as the reference diagnoses. As a number of studies continue to report quality problems in CR cause data, with a substantial problem of under-reporting HIV/AIDS,^13–17^^,^^43^^,^^44^ the VA diagnoses here were hence used as reference values in our study to assess the quality of the CR data.

Agreement of cause attribution was assessed with the kappa statistic and 95% confidence intervals (CIs) using the WHO List and short list. Misclassification patterns were identified by cross-tabulating the data using the short list. The sensitivity of the CR diagnoses and their positive predictive values (PPV) were calculated with 95% CIs. CSMFs were calculated as the proportion of total deaths attributable to specific conditions in each data set. The differences between CR- and VA-based fractions were expressed as percentages of the CR-based fractions, and 95% CIs were calculated using Nam and Blackwelder’s method.^45^

### Ethics

Ethics clearance for research involving human participants was obtained from the University of Queensland’s School of Population Health Research Ethics Committee (approval no. JJ010911), the South African Medical Research Council Ethics Committee (EC008-6/2011) and the Human Research Ethics Committee (Medical) at the University of the Witwatersrand (M120106). Ethical clearance for the collection of Agincourt HDSS and VA data was given by the University of the Witwatersrand Human Research Ethics Committee (Medical), clearance certificates M960720 and M110138.

## Results

### Data linkage

**Figure 2.**
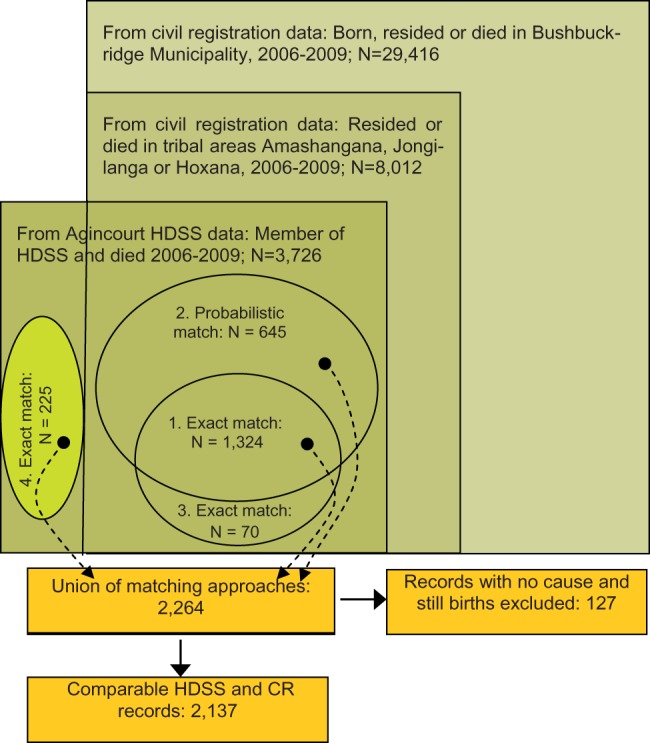
Establishing the study population: data sources, number of deaths and number of comparable records, Agincourt HDSS, 2006–09.

The complete CR data file with 4 years’ data contained 2 464 915 death records nationally. Of these, 29 416 records were found for individuals whose place of birth or residence or death was recorded as within the Bushbuckridge Municipality. These included 8012 records that had place of birth or residence or death recorded as within one of the three tribal areas in which the study site is located (Figure 2). The Agincourt HDSS data file contained 3726 death records of individuals who were residents of a household within the study site and died within the reference period. Deterministic and probabilistic record linkage approaches independently identified 1394 and 1969 matches, respectively, among records in the Agincourt HDSS and Stats SA’s CR data files where place of birth or residence or death in the CR data file was recorded as within the Bushbuckridge borders. Of the records that were matched with the deterministic approach, 1324 (95.0%) were also matched using the probabilistic approach. An additional 225 records were matched deterministically following careful examination of their variables and subsequent corrections to place names in the CR data, resulting in a total of 2264 matched cases (Figure 2). Over half of the deterministic matches (54%) were found via the deceased’s identity number. After removing 22 records of stillbirths and 105 with no VA cause of death recorded, 2137 records were available for assessing cause-of-death agreement.

Of all deaths recorded in the Agincourt HDSS database, 61% were matched to a death registered in the CR system. In contrast, for 85% of the Agincourt HDSS deaths, the household informant reported that the death had been registered at the Department of Home Affairs, the entity that administers the CR system. In both scenarios, the rate for deaths under the age of 5 years was substantially lower than that for persons aged over 5 years (Table 2).

**Table 2.** Matching rate and informant-reported level of death registration in the study population

Age	VA-CR matching rate			VA informant-reported registration		
	%	95% CI		%	95% CI	
All ages	60.8	59.2	62.3	84.6	83.4	85.8
<5 years	38.4	34.3	42.4	33.8	29.7	37.9
5+ years	64.7	63.0	66.3	93.3	92.4	94.2

### Characteristics of the study populations

The characteristics of the matched cases were fairly similar to those that did not match (Table 3 presents the 10 leading causes of death, according to the WHO List, prior to recoding and aggregating the causes into the short list. The VA system identified HIV disease as the leading cause of death (31%), in contrast to its 21st rank in the CR system (1.2%) (not presented). Diarrhoea and pneumonia are among the leading causes in both sources, but account for considerably higher proportions in the CR list. Injuries appear in both lists.

**Table 3.** Characteristics of the study populations, Agincourt HDSS and CR data, 2006–09

Characteristics	Agincourt HDSS matched		Agincourt HDSS unmatched		CR matched		CR unmatched	
	*n*	%	*n*	%	*n*	%	*n*	%
Age at death, years:								
<1	134	5.9	228	15.6	126	5.6	318	5.5
1–4	79	3.5	114	7.8	74	3.3	204	3.6
5–14	67	3.0	39	2.7	62	2.7	153	2.7
15–24	120	5.3	67	4.6	120	5.3	344	6.0
25–34	443	19.6	228	15.6	468	20.7	985	17.1
35–44	418	18.5	221	15.1	417	18.4	1045	18.2
45–54	284	12.5	169	11.6	271	12.0	760	13.2
55–64	229	10.1	125	8.6	227	10.0	551	9.6
65–74	203	9.0	95	6.5	207	9.1	526	9.2
75–84	171	7.6	109	7.5	183	8.1	544	9.5
85+	116	5.1	67	4.6	109	4.8	310	5.4
Unspecified	0	0.0	0	0.0	0	0.0	8	0.1
Total	2264	100.0	1462	100.0	2264	100.0	5748	100.0
Sex:								
Male	1160	51.2	795	54.4	1160	51.2	2774	48.3
Female	1104	48.8	667	45.6	1104	48.8	2964	51.6
Unspecified	0	0.0	0	0.0	0	0.0	10	0.2
Total	2264	100.0	1462	100.0	2264	100.0	5748	100.0
Venue of death:								
Hospital	1117	49.3	642	43.9	1058	46.7	2094	36.4
Heath care centre	25	1.1	17	1.1	–	–	–	–
Clinic	19	0.8	7	0.4	–	–	–	–
Emergency room/outpatient unit	–	–	–	–	37	1.6	95	1.7
Dead on arrival	–	–	–	–	9	0.4	52	0.9
Nursing home	–	–	–	–	6	0.3	30	0.5
Home	937	41.4	588	40.2	970	42.8	2784	48.4
Vehicle accident site	59	2.6	43	2.9	–	–	–	–
Other	98	4.3	157	10.7	50	2.2	206	3.6
Unknown/unassigned/unspecified	9	0.4	8	0.5	134	5.9	487	8.5
Total	2264	100.0	1462	100.0	2264	100.0	5748	100.0
Ascertainment of cause via:								
Verbal autopsy	2157	95.3	1346	92.1	–	–	–	–
Forensic autopsy	–	–	–	–	55	2.4	116	2.0
Opinion of attending doctor	–	–	–	–	717	31.7	1547	26.9
Opinion of attending doctor on duty	–	–	–	–	454	20.1	752	13.1
Opinion of registered nurse	–	–	–	–	5	0.2	4	0.1
Interview with family member	–	–	–	–	617	27.3	1814	31.6
Other	–	–	–	–	5	0.2	19	0.3
Unknown	–	–	–	–	28	1.2	55	1.0
Unspecified	107	4.7	116	7.9	383	16.9	1441	25.1
Total	2264	100.0	1462	100.0	2264	100.0	5748	100.0

), except that the unmatched compared with matched Agincourt HDSS cases had a higher proportion of child deaths under 5 years (23% vs 9%), and a higher proportion of cases with unspecified ascertainment of the cause of death (8% vs 5%). The age groups 25–34 and 35–44 years accounted for the largest proportions of deaths in both data sets.

### Leading underlying causes

Table 4 presents the 10 leading causes of death, according to the WHO List, prior to recoding and aggregating the causes into the short list. The VA system identified HIV disease as the leading cause of death (31%), in contrast to its 21st rank in the CR system (1.2%) (not presented). Diarrhoea and pneumonia are among the leading causes in both sources, but account for considerably higher proportions in the CR list. Injuries appear in both lists.

**Table 4.** Ten leading causes of deaths from VA and CR, according to the WHO List: Agincourt HDSS, 2006–09 (N = 2137)

Agincourt HDSS Verbal Autopsy (VA), according to WHO List			Civil Registration (CR), according to WHO List		
Rank	Ten leading causes	% of total	Rank	Ten leading causes	% of total
1	HIV disease (B20-B24)	31.4	1	Respiratory tuberculosis (A15-A16)	16.0
2	Respiratory tuberculosis (A15-A16)	14.8	2	Diarrhoea/gastro, infectious (A09)	15.6
3	Pneumonia (J12-J18)	5.2	3	Pneumonia (J12-J18)	10.7
4	Cerebrovascular diseases (I60-I69)	4.6	4	Cerebrovascular diseases (I60-I69)	5.2
5	Septicaemia (A40-A41)^a^	4.5	5	Other blood & immune disorders (D65-D89)	4.5
6	Meningitis (G00,G03)	3.8	6	All other external causes^b^	3.8
7	Diarrhoea/gastro, infectious (A09)	3.2	7	Other heart diseases (I26-I51)	3.6
8	Other heart diseases (I26-I51)	3.0	8	Hypertensive diseases (I10-I14)	3.0
9	Symptoms & ill-defined (R00-R99)	3.0	9	Other acute lower resp. infections (J20-J22)	3.0
10	Transport accidents (V01-V99)	2.4	10	Symptoms & ill-defined (R00-R99)	2.7
Top 10 causes as % of total deaths		76.1	Top 10 causes as % of total deaths		68.0

^a^Septicaemia is an intermediate cause of death, not an underlying cause of death. Although it is a clearly defined clinical entity, septicaemia has an underlying cause that would have precipitated the chain of events which led to death.^46^

^b^W20-W64, W75-W99, X10-X39, X50-X59, Y10-Y89.

### Cause agreement and misclassification

At the WHO list level, agreement of cause attribution between the VA and CR data was 15.1% (322/2137), yielding a kappa score of 0.1083 (95% CI: 0.0995 to 0.1171). At the much more aggregated short list level, agreement only increased to 23.2% (496/2137), kappa 0.1631 (CI: 0.1511 to 0.1751), or less than one in four cases.

Table 5 shows the misclassification patterns using the short list causes. Using the VA cause as reference diagnosis, the sensitivity of the CR system to identify external causes was relatively high (67%, 95% CI: 58.8 to 74.8), but considerably lower for natural causes. A relatively high PPV (78%, 95% CI: 69.7 to 85.0) was calculated for external causes, but noticeably lower values for natural causes.

**Table 5.** Misclassification patterns for selected causes/cause groups in the Agincourt HDSS study site, 2006–09

	Civil Registration diagnoses	Verbal Autopsy diagnoses																	
		Diarrhoea	Tuberculosis	HIV disease	Remaining infectious & parasitic disease	Neoplasms	Diabetes	Meningitis & meningococcal infection	Hypertensive disease	Remaining heart disease	Cerebrovascular disease	Acute lower respiratory infections	Other respiratory disease	Symptoms & ill{\hbox -}defined conditions	External causes	Remaining natural causes	CR total	Positive predictive value %	95% Confidence Interval
		1	2	3	4	5	6	7	8	9	10	11	12	13	14	15			
1	Diarrhoea	20	47	163	23	16	3	10	3	7	10	21	3	10	8	21	365	5.5	3.4 to 8.3
2	Tuberculosis	4	124	139	21	9	2	9	1	7	4	15	0	11	11	11	368	33.7	28.9 to 38.8
3	HIV disease	2	36	73	6	4	0	9	2	1	3	8	0	1	2	9	156	46.8	38.8 to 54.9
4	Remaining infect. & parasitic disease	4	6	17	13	3	0	3	1	1	1	3	0	2	5	6	65	20.0	11.1 to 31.8
5	Neoplasms	1	4	10	10	28	0	0	0	4	3	1	0	3	1	7	72	38.9	27.6 to 51.1
6	Diabetes	3	4	4	2	1	15	0	1	2	7	1	1	1	0	3	45	33.3	20.0 to 49.0
7	Meningitis	2	4	21	4	0	2	15	1	2	1	0	1	1	0	2	56	26.8	15.8 to 40.3
8	Hypertensive disease	7	4	7	6	6	2	4	4	9	4	7	1	1	0	2	64	6.3	1.7 to 15.2
9	Remaining heart disease	4	10	18	7	4	3	0	4	10	8	6	0	4	1	5	84	11.9	5.9 to 20.8
10	Cerebrovascular disease	2	3	10	14	5	3	5	10	4	34	10	1	7	0	3	111	30.6	22.2 to 40.1
11	Acute lower respiratory infections	10	44	118	20	9	2	14	7	7	8	21	1	10	7	15	293	7.2	4.5 to 10.7
12	Other respiratory disease	0	6	31	6	1	1	2	4	1	4	3	4	1	2	6	72	5.6	1.5 to 13.6
13	Symptoms & ill-defined conditions	4	13	15	11	4	1	5	0	3	4	8	0	4	3	4	79	5.1	1.4 to 12.5
14	External causes	1	2	2	1	1	0	4	0	2	2	0	1	7	96	4	123	78.0	69.7 to 85.0
15	Remaining natural causes	5	22	44	15	7	2	4	5	15	6	10	2	5	7	35	184	19.0	13.6 to 25.4
	VA total	69	329	672	159	98	36	84	43	75	99	114	15	68	143	133	2137		
	Sensitivity %	29.0	37.7	10.9	8.2	28.6	41.7	17.9	9.3	13.3	34.3	18.4	26.7	5.9	67.1	26.3			
	95% CI lower level	18.7	32.4	8.6	4.4	19.9	25.5	10.4	2.6	6.6	25.1	11.8	7.8	1.6	58.8	19.1			
	95% CI upper level	41.2	43.2	13.5	13.6	38.6	59.2	27.7	22.1	23.2	44.6	26.8	55.1	14.4	74.8	34.7			

The CR data show considerable misclassification of HIV disease. Of 672 VA deaths attributed to HIV disease (B20–B24), only 11% were assigned B20–B24 in the CR data (Table 5), and the remainder to 73 other ICD-10 codes. The most frequent single recipient CR causes were diarrhoea (24%), tuberculosis (20%) and pneumonia (13%), but heart failure (1.5%) and stroke (1.5%) were among the 10 leading recipient causes too. Of all recipient conditions, 21% were unexpected, including chronic respiratory diseases, peptic ulcers, diabetes, cardiac arrest and essential hypertension. Further, Table 5 suggests higher reporting of diabetes coupled with lower reporting of cardiovascular disease (CVD) deaths in the CR compared with VA diagnoses.

Table 6 shows the agreement characteristics and percent change in CSMFs if the underlying cause from the VA diagnosis replaced that from the CR diagnosis. The largest difference is observed for HIV, showing an expected 331% change. Significant differences are observed for most conditions, with only four conditions (tuberculosis, remaining heart disease, cerebrovascular disease and symptoms and ill-defined conditions) not significantly different.

**Table 6.** Agreement characteristics of civil registration and verbal autopsy diagnoses for the short list causes/cause groups: Agincourt HDSS, 2006–09

Conditions		Total occur-rences of the cause in CR system	On VA instrument				Cause-specific mortality fraction in CR data	Cause-specific mortality fraction in VA data	Per cent difference in cause-specific mortality fraction	95% CI
			Cause confirmed on VA instrument	Cause assigned to other causes in VA system	Received from other causes in VA system	Total occurrences of the cause in VA system				
1	Diarrhoea	365	20	345	49	69	17.1	3.2	−81	−85.2 to −75.9^a^
2	Tuberculosis	368	124	244	205	329	17.2	15.4	−11	−20.7 to 0.7
3	HIV disease	156	73	83	599	672	7.3	31.4	331	268.2 to 405.0^a^
4	Remaining infectious & parasitic disease	65	13	52	146	159	3.0	7.4	145	86.8 to 221.0^a^
5	Neoplasms	72	28	44	70	98	3.4	4.6	36	6.2 to 75.0^a^
6	Diabetes	45	15	30	21	36	2.1	1.7	−20	−43.8 to −13.3^a^
7	Meningitis	56	15	41	69	84	2.6	3.9	50	11.3 to 102.7^a^
8	Hypertensive disease	64	4	60	39	43	3.0	2.0	−33	−53.6 to −2.8^a^
9	Remaining heart disease	84	10	74	65	75	3.9	3.5	−11	−33.2 to 19.4
10	Cerebrovascular disease	111	34	77	65	99	5.2	4.6	−11	−28.7 to 14.2
11	Acute lower respiratory infections	293	21	272	93	114	13.7	5.3	−61	−68.3 to −52.3^a^
12	Other respiratory disease	72	4	68	11	15	3.4	0.7	−79	−87.7 to −65.0^a^
13	Symptoms & ill-defined conditions	79	4	75	64	68	3.7	3.2	−14	−37.1 to 17.8
14	External causes	123	96	27	47	143	5.8	6.7	16	2.4 to 32.6^a^
15	Remaining natural causes	184	35	149	98	133	8.6	6.2	−28	−40.7 to −12.0^a^
	Total	2137	496	1641	1641	2137	100.0	100.0		

^a^The confidence interval indicates significant changes in the cause-specific mortality fractions (*P* < 0.05).

### Injuries

Despite the relatively high sensitivity and PPV for external causes as a broad group, detailed analysis revealed very low sensitivity of the CR to ascertain suicide (0%; 0/23) and homicide (17%; 6/35). Ten deaths assigned unintentional injury codes in the CR system were attributed to intentional codes for suicide in the VA system. In the CR data, 76% of injuries (94/123) were assigned to ‘other', ‘unspecified' and undetermined-intent injury codes (V89, W76, X59, Y10-Y34). Most deaths in these groupings were assigned to more specific ICD-10 codes in the VA system (Table 7).

**Table 7.** External cause from VA for under-specified CR injury deaths, Agincourt HDSS 2006–09

Assigned in the Agincourt VA system		ICD-10 causes/cause groups assigned in CR system				VA total
ICD-10 cause category aggregated from single codes assigned	ICD-10 category code	Event of unde-termined intent: Y10-Y34	Exposure to unspecified factor: X59	Motor- or non-motor vehicle accident, vehicle unspecified: V89	Other accidental hanging and stran-gulation: W76	
Pedestrian injured in transport accident	V01 – V09	2	3	5	–	10
Car occupant injured in transport accident	V40 – V49	4	7	14	–	25
Occupant of pick-up truck or van injured in transport accident	V50 – V59	–	–	1	–	1
Other land transport accidents	V80 – V89	1	–	1	–	2
Falls	W00 – W19	–	–	1	–	1
Exposure to inanimate mechanical forces	W20 – W49	–	1	–	–	1
Exposure to smoke, fire and flames	X00 – X09	1	–	–	–	1
Exposure to forces of nature	X30 – X39	–	1	–	–	1
Intentional self-harm	X60 – X84	2	2	–	9	13
Assault	X85 – Y09	6	8	–	1	15
CR total		16	22	22	10	70

## Discussion

Cause-of-death agreement between the data sources was low, whether aggregated by the WHO (15.1%) or the short list (23.2%). Our study, and findings from urban^13^^,^^14^^,^^17^ and national^15^^,^^16^^,^^47^ studies, highlight systematic biases in CR cause-of-death data in South Africa. Similar to other research that indicates high proportions (73–92%) of HIV deaths being misattributed in the CR data,^14^^,^^16^^,^^28^ our study indicates misattribution in 89% of HIV deaths in the CR system. Yudkin *et al*.,^14^ for example, found tuberculosis, diarrhoea, lower respiratory and other respiratory infections, parasitic diseases, intestinal infectious diseases, meningitis, other infectious conditions, digestive disorders and ill-defined conditions as common HIV-recipient causes in their Cape Town study, comparing causes of death from CR and hospital records. Examining cause-of-death data from 1996–2001 CR records, Groenewald *et al*.^15^ found that the following conditions increased in the same distinct age pattern as HIV/AIDS and concluded that they could be considered misattributed HIV/AIDS deaths: tuberculosis, pneumonia, diarrhoea, meningitis, other respiratory disease, non-infective gastroenteritis, other infectious and parasitic diseases, deficiency anaemia and protein energy malnutrition. These findings are resonated in a post-mortem autopsy study in a tertiary hospital in rural Eastern Cape province of South Africa, showing that the leading three causes of death among HIV-positive people were tuberculosis, pneumonia and meningitis.^48^ Similarly, acute respiratory infections and tuberculosis were among the most frequent causes of death assigned to deaths among HIV-infected persons in a study pooling data from six sites of the Alpha Network in Africa.^49^ Such misattribution, and the resultant underreporting of deaths from HIV/AIDS, have been attributed to cause-of-death coding practices, legal issues regarding life and health insurance, concerns regarding the confidentiality of death certificates, the fear of HIV-positive stigmatization, and clinicians avoiding HIV/AIDS on the death certificate because of the potential for harm.^14–16^^,^^50–52^ Whereas misattribution of HIV to infectious conditions such as diarrhoea, tuberculosis and pneumonia has been acknowledged in other studies^14–16^ and account for the majority HIV-recipient conditions in our study, our findings highlight that HIV deaths were also misattributed to selected circulatory diseases, neoplasms and digestive, endocrine and metabolic disorders. Previously-observed misclassification patterns between diabetes and CVD are also confirmed.^13^^,^^47^ These misclassifications and biases indicate that the CR data cannot be taken at face value and caution should be exercised in its use for research and health decision-making.

Compared with natural causes of death, better agreement was observed for external causes as a group. However, detailed cause analysis indicates that the CR diagnoses missed the majority of homicide and all suicide cases, and that the external causes for three-quarters of injury deaths remain undetermined. This is a consequence of the death notification form (DNF) not including a field for intent of injury deaths (homicide/suicide/accident/unknown). The manner of death is consequently often missing and results in the statistics having limited capacity to guide injury prevention or safety promotion interventions.

Surprisingly, the proportion of causes coded to non-specific and ill-defined causes for this area was low (3–4%, compared with about 14% nationally for the same period).^31^ R-codes in the relevant province, Mpumalanga, were also low (9%) in the period 1997–2007, compared with neighbouring Gauteng (12%), KwaZulu-Natal (15%) and Limpopo (18%).^9^ One possible explanation is that headmen do not certify deaths in the Agincourt study site, but further research into local certification practices is required to explain the phenomenon.

Considering the cause-of-death profile from both data sources, and acknowledging misattribution and undereporting of HIV/AIDS, it is clear that HIV/TB is a major concern in the area. In addition, the data suggest the emergence of cardiovascular disease as an epidemiological concern. Though not a vote of confidence in the CR cause-of-death certification system, this broad similarity suggests that, despite low agreement of cause attribution at the individual level, there is scope to carefully interpret and adjust CR data to identify plausible broad epidemiological patterns at the population level in rural areas, to prioritize health care needs and inform public health policy. It must be noted that the CR data, however, could be misleading with respect to specific causes.

It was not possible to apply the capture-recapture method to calculate CR completeness of death registration because the study site boundaries differed from the official boundaries used in CR data. This resulted in challenges to identify whether a CR death occurred within the HDSS borders. Further challenges arose from the fact that a tribal area name instead of the actual village name was sometimes recorded on the DNF. Each tribal area spans an area larger than the village areas and, combined, the tribal areas span an area larger than the study site (Figure 1). Additionally, the study identified some differences in the place names of villages used by the Agincourt HDSS and StatsSA, indicating an inability to validate the colloquial name against the official CR name. Valuable lessons were learned about the need for accurate place name reporting during death reporting, place name consistency across data sources, and the alignment of study-site and official boundaries.

Although we could not calculate completeness of death registration using a standard capture-recapture approach, the matching rate of 61% could be taken as a minimum indication of completeness. However, the matching rate was adversely affected by various limiting factors, including few common variables to work with and proxy reporting of vital-event information.^36^^,^^53^ Given these limiting factors, it is likely that completeness was higher than the matching rate. This likelihood is supported by the VA-informant responses indicating that 85% of deaths were registered into the CR system. As socially desirable answers and recall limitations both are acknowledged sources of bias that may affect informant responses, further research is needed to better inform our understanding of completeness in the area.

Accurate cause-of-death attribution, including that for HIV/AIDS, is needed in order for CR to meet its potential to directly inform decision-making in South Africa and contribute to regional estimates. There is an urgent need to improve the quality of CR cause-of-death information, with renewed and innovative efforts. It is encouraging that Stats SA, together with the Department of Health, Department of Home Affairs and Medical Research Council have set up a training initiative to improve the quality of cause-of-death certification countrywide.^51^ In addition, an independent, systematic, scientific effort, such as a national burden of disease study utilizing all mortality data sources to identify data problems and adjust for biases, will provide valuable estimates. However, there is a need to consider further actions to address the biases. A nationally representative validation study of CR cause data against ‘gold standard’ instruments, such as post-mortem autopsy reports or high-quality laboratory, hospital and other medical records, would likely boost confidence in the country’s CR data. Improving the quality of medical records, however, is likely a prerequisite.^24^^,^^44^ For substantial improvements to injury data to be made, the Department of Home Affairs is urged to include a field for intent, or apparent manner of death, in a revised DNF, to ensure that details about external causes, needed for prevention efforts, can be reported. Finally, to address the gap arising from poor cause specification for home deaths, we suggest that registration of these deaths in the CR system be augmented by the systematic collection of VAs to support the current practices for physician certification at morgues. Further, systematic VAs could also be used in those deaths certified by village headmen. The human capacity for such an initiative is potentially available within the community health worker programme being established as part of primary health care re-engineering. This, and harnessing the many years of experience and strengths from the country’s three HDSSs, would provide a critical mass of human resources and training capacity to strengthen cause-of-death ascertainment through the use of VAs for out-of-hospital deaths. The WHO short-form VA questionnaire^54^ and promising automated methods^49^^,^^55–58^ will greatly facilitate the application of VA in routine CR systems for diagnosing out-of-hospital deaths, including in South Africa.

This first study linking national CR and HDSS data demonstrates that data linkage between these sources is possible. This offers a promising method to provide empirical evidence about the quality and utility of rural CR mortality data, and show how matching can be used to better understand, complement and improve the quality of CR mortality data. In addition, the study offers the first empirical evidence of the extent and diversity of misattribution of HIV deaths in a rural setting. Previously, such inferences were based on indirect evidence such as the implausibility of age distributions for HIV-recipient/indicator conditions. The Agincourt HDSS data hold considerable value in providing much-needed detail on external causes of injury deaths to better inform programmes aimed at reducing fatal injuries. Urban and national findings of systematic biases are confirmed by this rural study, pointing to the countrywide urgency to improve CR cause-of-death data. In the interim, however, it is encouraging that the confirmed biases can facilitate adjustment of cause profiles after careful interpretation, to better inform rural health prioritization and planning.

## Funding

This work was supported by: the Medical Research Council/Wits University/Rural Public Health and Health Transitions Research Unit (Agincourt); the South African Medical Research Council; and Statistics South Africa. The research was carried out while the first author held a University of Queensland Research Scholarship and the Endeavour International Postgraduate Research Scholarship at the University of Queensland, Brisbane, Australia. The Agincourt HDSS was funded by: the Wellcome Trust, UK (grants no. 058893/Z/99/A, 069683/Z/02/Z, 085477/Z/08/Z); the National Institute on Ageing of the NIH (grants 1R24AG032112-01 and 5R24AG032112-03); the William and Flora Hewlett Foundation (grant 2008-1840); the Andrew W Mellon Foundation, USA; and the University of the Witwatersrand and Medical Research Council, South Africa. The funders had no role in study design, data collection, analysis, decision to publish or preparation of the manuscript.

## Author contributions

A.D.L., C.R., D.B., T.V. and J.J. conceptualized the study. J.J. wrote the study proposal and ethics applications; coordinated the collaboration, data preparation and matching exercise; conceptualized the paper; analysed and interpreted the data; created the tables and graphs; wrote the first draft of all sections of the paper; integrated inputs from co-authors; submitted the paper; and led the response to reviewers’ comments. K.K. was instrumental in the ethics application process at the University of the Witwatersrand. A.L., D.B., K.K., S.T. and T.V. made substantial contributions to negotiating access to and procuring the data. C.K. extracted the Agincourt HDSS data with inputs from K.K., P.M. and S.T. C.K. did the electronic matching and created the base analytic data set with inputs from D.B., J.J. and T.V. C.R. revised the manuscript critically for structure and word economy, critically appraised the data visualizations and reviewed the text for important intellectual content. A.L., C.R., D.B. and T.V. consistently supplied detailed comments during all phases of the study and critically appraised decisions regarding the methods, findings and interpretation of the results. All authors made contributions to the design of the study, interpretation of the data and critical review of the final manuscript, and approved the final version to be published.
